# Fat‐to‐muscle ratio as a predictor of insulin resistance and metabolic syndrome in Korean adults

**DOI:** 10.1002/jcsm.12548

**Published:** 2020-02-07

**Authors:** Young‐Gyun Seo, Hong Ji Song, Young Rim Song

**Affiliations:** ^1^ Department of Family Medicine Hallym University Sacred Heart Hospital Anyang Gyeonggi‐do Republic of Korea; ^2^ Department of Internal Medicine Hallym University Sacred Heart Hospital Dongan‐gu Anyang Gyeonggi‐do Republic of Korea

**Keywords:** Sarcopenia, Muscle, Lean mass, Fat‐to‐muscle ratio, Insulin resistance, Metabolic syndrome

## Abstract

**Background:**

The present study evaluated the associations of the fat‐to‐muscle ratio (FMR) with metabolic syndrome (MetS) and insulin resistance (IR) in Korean adults using nationally representative survey data.

**Methods:**

A two‐stage stratified sampling method was reflected in a cross‐sectional study involving a total of 13 032 participants aged ≥ 19 years who participated in the fourth and fifth Korea National Health and Nutrition Examination Surveys. The homeostasis model assessment for IR (HOMA‐IR) was used to evaluate IR and was calculated as follows: [fasting plasma glucose level (mg/dL) × fasting plasma insulin level (uIU/mL)]/405. MetS was defined using the 2006 International Diabetes Federation criteria, and FMR was measured using whole‐body dual‐energy X‐ray absorptiometry and calculated as follows: total fat mass (kg) divided by total lean mass (kg). In addition, the optimal FMR cut‐off values for detecting MetS and the odds ratios (ORs) for MetS risk were determined according to the FMR quartile and sex.

**Results:**

Among all participants, the proportion of women was 58.4%, and the mean age was 44.22 ± 0.26 years. The FMR significantly differed between men and women (0.30 ± 0.002 vs. 0.53 ± 0.003, respectively, *P* < 0.001), and the prevalence of MetS and IR gradually increased as FMR increased (*P* for trend: <0.001). The optimal FMR cut‐off value for detecting MetS was higher in women than in men (0.555 vs. 0.336, respectively). The negative predictive value was the highest in normal‐weight participants (0.9992 in women and 0.9986 in men), while the positive predictive value was the highest in obese participants (0.5994 in women and 0.5428 in men). Based on the derived cut‐off FMR, a high FMR was associated with poor outcomes in terms of cardiometabolic risk markers (*P* < 0.001). The multivariable‐adjusted ORs for MetS, abdominal obesity, and IR (HOMA‐IR ≥ 3) were 5.35 [95% confidence interval (CI): 4.39–6.52], 7.67 (95% CI: 6.33–9.30), and 3.25 (95% CI: 2.70–3.92), respectively, in men and 5.59 (95% CI: 4.66–6.72), 7.48 (95% CI: 6.35–8.82), and 2.55 (95% CI: 2.17–3.00), respectively, in women.

**Conclusions:**

In the present study, a high FMR was significantly associated with the prevalence of MetS and IR. The present findings also showed that FMR can be a novel indicator for detecting the absence or presence of MetS, particularly in metabolically healthy normal‐weight individuals and metabolically obese obese‐weight individuals.

## 1 Introduction

Obesity has become a major health problem, owing to its increasing prevalence worldwide, and is an important risk factor for metabolic syndrome (MetS) and cardiovascular diseases.^1^, ^2^, ^3^ Body mass index (BMI) is the most commonly used method to assess overweightness and obesity, which are characterized by excessive accumulation of fat. However, BMI has several limitations in assessing obesity‐related cardiometabolic risks because it cannot distinguish between fat and muscle mass.^4^, ^5^ For example, normal‐weight obesity can be associated with MetS and insulin resistance (IR),^6^, ^7^ and metabolically obese normal‐weight individuals are associated with increased cardiovascular risks compared with metabolically healthy obese individuals.^8^, ^9^

Furthermore, Asians suffer from obesity‐related complications at lower BMI values than do Caucasians,^10^, ^11^ and individuals in the same BMI category can exhibit heterogeneous metabolic and functional characteristics such as differences in blood pressure (BP), lipid profiles, glucose intolerance, physical activity (PA), muscle mass, and visceral obesity.^5^, ^7^, ^12^, ^13^ Because the close correlation between abdominal fat and MetS has been validated by many studies, waist circumference (WC) is now used as one of the criteria included in the diagnosis of MetS.^14^, ^15^, ^16^, ^17^ However, there are some limitations to using WC to assess the risk of obesity; for example, there are population‐specific cut‐off levels for abdominal obesity.^18^ In addition, visceral adipose tissue is pro‐inflammatory^19^ and has been associated with cardiometabolic risks across BMI categories. Thus, visceral adiposity could represent a more sensitive index of cardiometabolic risk when combined with measures of low muscle mass.^20^, ^21^ In addition, WC does not accurately reflect visceral adiposity, and thus imaging studies are needed to ensure accurate evaluations. However, the regular assessment of visceral fat using computed tomography, magnetic resonance imaging, and/or abdominal ultrasounds remains limited owing to the high costs and/or radiation exposure.

Some studies have proposed that body composition measurements are useful for assessing cardiometabolic risk.^22^, ^23^, ^24^, ^25^, ^26^ For example, low muscle mass and high fat mass (FM) may play important roles in MetS and cardiovascular diseases,^20^, ^21^, ^27^ because FM is considered an appropriate marker of total body fat and improves the predictive power of MetS.^22^ Recently, use of the fat‐to‐muscle ratio (FMR) was introduced as a novel assessment of the combined effects of fat and skeletal muscle mass. The FMR is associated with IR, liver fat accumulation, and MetS,^28^, ^29^, ^30^, ^31^, ^32^, ^33^, ^34^ and our research group has reported that the FMR is a useful indicator of metabolic and inflammatory statuses, as well as an independent risk factor for the long‐term outcomes of patients with chronic kidney disease.^32^ A previous study has explored FMR thresholds for diagnosing MetS using bioelectrical impedance analysis (BIA) in young adults.^28^ Therefore, the objective of this study was to evaluate the associations of FMR with MetS and IR according to obesity status using whole‐body dual‐energy X‐ray absorptiometry (DXA) in nationally representative survey data including Korean adults of all ages.

## 2 Methods

### 2.1 Study participants

The present study evaluated data from the fourth and fifth Korea National Health and Nutrition Examination Surveys (KNHANES), which is a nationally representative survey conducted between 2008 and 2011 by the Korea Centers for Disease Control and Prevention (KCDC). To assess the relationship between FMR and cardiometabolic risk, the data from 18 915 participants aged ≥ 19 years who underwent DXA were examined. Participants who had severely reduced kidney function (estimated glomerular filtration rate < 30; *n* = 82), had ever been diagnosed with cancer (*n* = 598), fasted for >24 or <8 h (*n* = 638) prior to the health examination, had an inadequately low or high daily energy intake (<500 or >5000 kcal/day, respectively; *n* = 217), had an inadequately high daily water intake per body weight (≥90 g/kg; *n* = 5), and/or had missing survey records or examination results (*n* = 4343) were excluded from the analyses. Ultimately, 13 032 participants were selected for inclusion in the present analyses.

All procedures have been approved by the ethics committee of the KCDC (Institutional Review Board numbers: 2008‐04EXP‐01‐C, 2009‐01CON‐03‐2C, 2010‐02CON‐21‐C, and 2011‐02CON‐06‐C) and have therefore been performed in accordance with the ethical standards laid down in the 1964 Declaration of Helsinki and its later amendments. Signed informed consent was obtained from all KNHANES participants, and because the KNHANES data are publicly available, no further institutional review board approval was required for the study protocol.

### 2.2 Fat‐to‐muscle ratio

Body composition was measured using whole‐body DXA (Discovery, Hologic Inc.; Bedford, MA, USA) by licensed and trained technicians. Data from DXA included values for bone mineral content (g), bone mineral density (g/cm^2^), FM (g), lean mass (g), total mass (g), and fat percentage (%) of the whole body and six regions (head, left arm, right arm, trunk, left leg, and right leg). The FMR was calculated as the whole‐body FM divided by the whole‐body lean mass (bone mineral contents were subtracted) and was grouped into quartiles (Q1–Q4) from the lowest (Q1) to highest (Q4) values.

### 2.3 Metabolic syndrome

The 2001 National Cholesterol Education Program/Adult Treatment Panel III^35^ and 2005 American Heart Association/National Heart, Lung, and Blood Institute^36^ criteria were used to define MetS on the basis the presence of any three of the following five traits: (i) WC ≥ 90 cm in men and ≥85 cm in women, which are the WC cut‐off levels for abdominal obesity in Koreans^37^; (ii) serum triglyceride (TG) level ≥ 150 mg/dL or receiving drug treatment for dyslipidaemia; (iii) serum high‐density lipoprotein (HDL) cholesterol level < 40 mg/dL in men or <50 mg/dL in women; (iv) BP ≥ 130/85 mmHg or receiving drug treatment for elevated BP; and (v) fasting plasma glucose (FPG) level ≥ 100 mg/dL or receiving drug treatment for elevated blood glucose levels.

The 2006 International Diabetes Federation (IDF)^18^ criteria were used to define MetS on the basis of the presence of abdominal obesity (WC ≥ 90 cm in men and ≥85 cm in women) plus any two of the following four traits: (i) TG level ≥ 150 mg/dL or receiving treatment for dyslipidaemia; (ii) HDL cholesterol level < 40 mg/dL in men or <50 mg/dL in women; (iii) systolic BP ≥ 130, diastolic BP ≥ 85, or receiving treatment for hypertension; and (iv) FPG level ≥ 100 mg/dL or previously diagnosed with type 2 diabetes.

### 2.4 Insulin resistance

The homeostasis model assessment for IR (HOMA‐IR) was used to evaluate IR and was calculated using the following formula: [FPG level (mg/dL) × fasting plasma insulin level (uIU/mL)]/405.

### 2.5 Obesity status

The World Health Organization BMI cut‐off levels in adult Asians are 18.5, 23, and 25 kg/m^2,^ 38 with underweight, normal weight, overweight, and obesity defined as BMI < 18.5, 18.5–22.9, 23–24.9, and ≥25 kg/m^2^, respectively.

### 2.6 Variables

For sex‐based analyses, the following data were collected: age, FMR, WC, MetS status, HOMA‐IR, BMI, obesity status, daily nutritional intake (total energy intake; percentages of energy intake from carbohydrate, protein, and fat; and water intake per body weight by the 24 h recall method), smoking status (none, past, or current), monthly alcohol consumption (<once or ≥once/month), education level (≤elementary school, middle or high school, and ≥college), average monthly household income (quartile), co‐morbidity (hypertension, diabetes, or dyslipidaemia), and survey year. PA was assessed as the metabolic equivalent value or as a categorical variable (low, moderate, or high) on the basis of the International Physical Activity Questionnaire data processing and analysis guidelines.^39^

### 2.7 Statistical analysis

KNHANES data were extracted using two‐stage stratified cluster sampling rather than simple random sampling; therefore, the complex sampling weights are reflected in the present data analyses. Linear regression analyses and *χ* ^2^ tests were conducted to compare the general characteristics among the FMR quartiles according to sex, and then box plots of the FMR according to sex and age were drawn. In addition, the relationship between FMR and IR was analysed by estimating the least square means (marginal means) of the HOMA‐IR according to FMR quartile. Logistic regression analyses were performed to assess MetS risk according to FMR quartile and to compare daily nutritional intake levels according to FMR quartile. Adjusted odds ratios (ORs) or beta coefficients were calculated after accounting for potential confounding variables such as age, total energy intake, water intake per body weight, smoking status, monthly alcohol consumption, PA, education level, average monthly household income, and survey year.

A receiver operating characteristic (ROC) curve adjusted for potential confounding variables was constructed, using the point on the ROC curve closest to (0, 1) as a criterion to calculate the cut‐off FMR for assessing MetS risk according to obesity status. We also calculated the cut‐off FMR using criterion based on Youden's index. We obtained potential cut‐offs from the minimum distance criterion and adopted the cut‐off that maximized Youden's index. We also examined the sensitivity, specificity, likelihood ratio positive and negative, and positive and negative predictive values. After the participants were divided according to the cut‐off FMR, cardiometabolic risk levels were compared between groups using the linear regression analysis and the *χ* ^2^ test. In addition, logistic regression analysis was performed to compare cardiometabolic risk levels using the cut‐off FMR for MetS. All statistical analyses were conducted using Stata/MP version 14.0 (StataCorp., College Station, TX, USA). All statistical tests were two‐sided, and a *P* value < 0.05 was considered to indicate statistical significance.

## 3 Results

### 3.1 General characteristics

Of the 13 032 participants in the present study, 58.4% were women, and the mean age was 44.22 ± 0.26 years. *Table* 1 shows the general characteristics of the participants among the FMR quartiles according to sex. As FMR increased, WC, BMI, and HOMA‐IR gradually increased; and co‐morbidity prevalence, MetS prevalence, and the proportion of participants satisfying each MetS criterion tended to gradually increase. In FMR Q4, participants with a WC ≥ 90 cm accounted for >50%; those with TG ≥ 150 mg/dL or drug treatment for dyslipidaemia accounted for close to 50%; and those with HDL < 40 mg/dL, BP ≥ 130/85 mmHg or drug treatment for an elevated BP, and FPG ≥ 100 mg/dL or drug treatment for an elevated FPG accounted for >40%. On the other hand, in FMR Q1, the proportion of participants satisfying each MetS criterion was <25%. However, the proportion of normal‐weight participants tended to gradually decrease as FMR increased. In FMR Q3, men with a percentage body fat (%BF) of ≥25% accounted for <20%, while women with a %BF ≥ 35% accounted for close to 50%. FMR Q4 had the lowest proportion of current smokers, but the highest proportion of past smokers. FMR Q4 had the lowest proportion of alcohol drinkers (≥once/month), while FMR Q3 had the highest proportion of alcohol drinkers (≥once/month). In addition, FMR Q4 had the lowest proportion of men with a high PA level.

**Table 1:** General characteristics of the study participants

Sex	Characteristics		Total	Q1	Q2	Q3	Q4	*P* value^a^	Post‐hoc test^b^
Male			(*n* = 5425)	(*n* = 1357)	(*n* = 1356)	(*n* = 1356)	(*n* = 1356)		
	Age, years		44.03 ± 0.33	41.70 ± 0.60	44.84 ± 0.49	45.39 ± 0.52	44.29 ± 0.54	0.000	Q1 < Q2, Q3, Q4
	Fat‐to‐muscle ratio		0.30 ± 0.002	0.19 ± 0.001	0.27 ± 0.001	0.33 ± 0.001	0.43 ± 0.003	0.000	Q1 < Q2 < Q3 < Q4
	Waist circumference, cm		83.98 ± 0.18	75.91 ± 0.25	82.86 ± 0.25	86.30 ± 0.25	91.39 ± 0.36	0.000	Q1 < Q2 < Q3 < Q4
	Body mass index, kg/m^2^		24.04 ± 0.06	21.40 ± 0.08	23.60 ± 0.08	24.69 ± 0.09	26.63 ± 0.13	0.000	Q1 < Q2 < Q3 < Q4
	Obesity status							0.000	
		Underweight	180 (3.32)	160 (11.79)	15 (1.11)	4 (0.29)	1 (0.07)		
		Normal	1907 (35.15)	883 (65.07)	540 (39.82)	331 (24.41)	153 (11.28)		
		Overweight	1416 (26.10)	213 (15.70)	451 (33.26)	444 (32.74)	308 (22.71)		
		Obesity	1922 (35.43)	101 (7.44)	350 (25.81)	577 (42.55)	894 (65.93)		
	Percentage body fat							0.000	
		<25%	3814 (70.30)	1357 (100.00)	1356 (100.00)	1101 (81.19)	0 (0.00)		
		≥25%	1611 (29.70)	0 (0.00)	0 (0.00)	255 (18.81)	1356 (100.00)		
	Nutritional intake								
		Total energy, kcal/day	2331.98 ± 15.05	2397.76 ± 30.81	2356.31 ± 28.81	2341.34 ± 29.59	2227.38 ± 28.23	0.000	Q1, Q2, Q3 > Q4
		Carbohydrate, % of energy	62.99 ± 0.25	62.99 ± 0.48	63.76 ± 0.44	62.24 ± 0.50	62.94 ± 0.47	0.429	
		Protein, % of energy	14.51 ± 0.07	14.30 ± 0.13	14.42 ± 0.14	14.64 ± 0.15	14.68 ± 0.14	0.028	Q1 < Q4
		Fat, % of energy	18.03 ± 0.16	17.93 ± 0.28	17.48 ± 0.30	18.52 ± 0.30	18.18 ± 0.28	0.154	
		Water intake/body weight, g/kg/day	16.41 ± 0.17	17.86 ± 0.33	16.95 ± 0.33	16.26 ± 0.37	14.46 ± 0.32	0.000	Q1 > Q2, Q3 > Q4
	Smoking							0.000	
		None	1219 (22.47)	329 (24.24)	299 (22.05)	278 (20.50)	313 (23.08)		
		Past	1989 (36.66)	387 (28.52)	511 (37.68)	539 (39.75)	552 (40.71)		
		Current	2217 (40.87)	641 (47.24)	546 (40.27)	539 (39.75)	491 (36.21)		
	Alcohol consumption							0.006	
		<1 time/month	1377 (25.38)	350 (25.79)	337 (24.85)	310 (22.86)	380 (28.02)		
		≥1 time/month	4048 (74.62)	1007 (74.21)	1019 (75.15)	1046 (77.14)	976 (71.98)		
	Physical activity (IPAQ)^c^							0.000	
		Low	1501 (27.67)	299 (22.03)	365 (26.92)	398 (29.35)	439 (32.37)		
		Moderate	2101 (38.73)	488 (35.96)	497 (36.65)	548 (40.41)	568 (41.89)		
		High	1823 (33.60)	570 (42.00)	494 (36.43)	410 (30.24)	349 (25.74)		
	Education							0.000	
		≤Elementary school	860 (15.85)	252 (18.57)	226 (16.67)	215 (15.86)	167 (12.32)		
		Middle or high school	2278 (41.99)	587 (43.26)	586 (43.22)	559 (41.22)	546 (40.27)		
		≥College	2287 (42.16)	518 (38.17)	544 (40.12)	582 (42.92)	643 (47.42)		
	Income							0.000	
		Q1	1277 (23.54)	374 (27.56)	336 (24.78)	292 (21.53)	275 (20.28)		
		Q2	1413 (26.05)	350 (25.79)	336 (24.78)	342 (25.22)	385 (28.39)		
		Q3	1363 (25.12)	346 (25.50)	348 (25.66)	338 (24.93)	331 (24.41)		
		Q4	1372 (25.29)	287 (21.15)	336 (24.78)	384 (28.32)	365 (26.92)		
	Co‐morbidity								
		Hypertension	1209 (22.29)	168 (12.38)	288 (21.24)	328 (24.19)	425 (31.34)	0.000	
		Diabetes	457 (8.42)	70 (5.16)	114 (8.41)	141 (10.40)	132 (9.73)	0.000	
		Dyslipidaemia	445 (8.20)	46 (3.39)	104 (7.67)	132 (9.73)	163 (12.02)	0.000	
	Survey year							0.000	
		2008	940 (17.33)	319 (23.51)	248 (18.29)	213 (15.71)	160 (11.8)		
		2009	2127 (39.21)	574 (42.30)	565 (41.67)	534 (39.38)	454 (33.48)		
		2010	1681 (30.99)	303 (22.33)	355 (26.18)	436 (32.15)	587 (43.29)		
		2011	677 (12.48)	161 (11.86)	188 (13.86)	173 (12.76)	155 (11.43)		
	MetS component								
		WC ≥ 90 cm	1428 (26.32)	37 (2.73)	197 (14.53)	443 (32.67)	751 (55.38)	0.000	
		TG ≥ 150 mg/dL or drug treatment for dyslipidaemia	1994 (36.76)	234 (17.24)	479 (35.32)	614 (45.28)	667 (49.19)	0.000	
		HDL < 40 mg/dL	1830 (33.73)	250 (18.42)	446 (32.89)	543 (40.04)	591 (43.58)	0.000	
		BP ≥ 130/85 mmHg or drug treatment for elevated BP	1807 (33.31)	310 (22.84)	466 (34.37)	485 (35.77)	546 (40.27)	0.000	
		FPG ≥ 100 mg/dL or drug treatment for elevated FPG	1798 (33.14)	274 (20.19)	474 (34.96)	493 (36.36)	557 (41.08)	0.000	
	MetS (NCEP)		1435 (26.45)	101 (7.44)	313 (23.08)	430 (31.71)	591 (43.58)	0.000	
	MetS (IDF)		915 (16.87)	26 (1.92)	135 (9.96)	281 (20.72)	473 (34.88)	0.000	
	HOMA‐IR		2.42 ± 0.03	1.83 ± 0.03	2.24 ± 0.04	2.55 ± 0.05	3.08 ± 0.09	0.000	Q1 < Q2 < Q3 < Q4

Female			(*n* = 7607)	(*n* = 1902)	(*n* = 1902)	(*n* = 1902)	(*n* = 1901)		
	Age, years		44.40 ± 0.28	40.22 ± 0.45	43.66 ± 0.42	46.50 ± 0.46	47.54 ± 0.55	0.000	Q1 < Q2 < Q3, Q4
	Fat‐to‐muscle ratio		0.53 ± 0.003	0.37 ± 0.001	0.48 ± 0.001	0.57 ± 0.001	0.70 ± 0.003	0.000	Q1 < Q2 < Q3 < Q4
	Waist circumference, cm		77.62 ± 0.18	70.02 ± 0.24	75.60 ± 0.23	79.85 ± 0.27	85.62 ± 0.35	0.000	Q1 < Q2 < Q3 < Q4
	Body mass index, kg/m^2^		23.19 ± 0.06	20.35 ± 0.07	22.35 ± 0.07	23.90 ± 0.08	26.37 ± 0.13	0.000	Q1 < Q2 < Q3 < Q4
	Obesity status							0.000	
		Underweight	408 (5.36)	336 (17.67)	47 (2.47)	21 (1.10)	4 (0.21)		
		Normal	3374 (44.35)	1314 (69.09)	1093 (57.47)	683 (35.91)	284 (14.94)		
		Overweight	1672 (21.98)	189 (9.94)	489 (25.71)	566 (29.76)	428 (22.51)		
		Obesity	2153 (28.30)	63 (3.31)	273 (14.35)	632 (33.23)	1185 (62.34)		
	Percentage body fat							0.000	
		<35%	4845 (63.69)	1902 (100.00)	1902 (100.00)	1041 (54.73)	0 (0.00)		
		≥35%	2762 (36.31)	0 (0.00)	0 (0.00)	861 (45.27)	1901 (100.00)		
	Nutritional intake								
		Total energy, kcal/day	1673.49 ± 9.70	1733.33 ± 20.12	1693.64 ± 18.56	1654.48 ± 17.07	1607.61 ± 17.71	0.000	Q1 > Q3 > Q4; Q2 > Q4
		Carbohydrate, % of energy	68.72 ± 0.20	68.05 ± 0.35	68.74 ± 0.36	69.28 ± 0.35	68.84 ± 0.38	0.056	
		Protein, % of energy	14.21 ± 0.06	14.12 ± 0.11	14.22 ± 0.12	14.23 ± 0.11	14.29 ± 0.11	0.270	
		Fat, % of energy	17.17 ± 0.14	17.96 ± 0.27	17.32 ± 0.26	16.43 ± 0.23	16.90 ± 0.29	0.001	Q1, Q2 > Q3; Q1 > Q4
		Water intake/body weight, g/kg/day	15.11 ± 0.16	17.25 ± 0.31	15.82 ± 0.27	14.56 ± 0.26	12.63 ± 0.23	0.000	Q1 > Q2 > Q3 > Q4
	Smoking							0.007	
		None	6957 (91.46)	1715 (90.17)	1754 (92.22)	1738 (91.38)	1750 (92.06)		
		Past	284 (3.73)	69 (3.63)	58 (3.05)	84 (4.42)	73 (3.84)		
		Current	366 (4.81)	118 (6.20)	90 (4.73)	80 (4.21)	78 (4.10)		
	Alcohol consumption							0.000	
		<1 time/month	4568 (60.05)	1074 (56.47)	1091 (57.36)	1186 (62.36)	1217 (64.02)		
		≥1 time/month	3039 (39.95)	828 (43.53)	811 (42.64)	716 (37.64)	684 (35.98)		
	Physical activity (IPAQ)^c^							0.097	
		Low	2489 (32.72)	606 (31.86)	600 (31.55)	624 (32.81)	659 (34.67)		
		Moderate	3267 (42.95)	792 (41.64)	830 (43.64)	835 (43.90)	810 (42.61)		
		High	1851 (24.33)	504 (26.50)	472 (24.82)	443 (23.29)	432 (22.72)		
	Education							0.000	
		≤Elementary school	2149 (28.25)	423 (22.24)	464 (24.40)	600 (31.55)	662 (34.82)		
		Middle or high school	3071 (40.37)	711 (37.38)	810 (42.59)	770 (40.48)	780 (41.03)		
		≥College	2387 (31.38)	768 (40.38)	628 (33.02)	532 (27.97)	459 (24.15)		
	Income							0.449	
		Q1	1844 (24.24)	477 (25.08)	457 (24.03)	446 (23.45)	464 (24.41)		
		Q2	1954 (25.69)	480 (25.24)	468 (24.61)	504 (26.50)	502 (26.41)		
		Q3	1950 (25.63)	477 (25.08)	525 (27.60)	480 (25.24)	468 (24.62)		
		Q4	1859 (24.44)	468 (24.61)	452 (23.76)	472 (24.82)	467 (24.57)		
	Co‐morbidity								
		Hypertension	1439 (18.92)	160 (8.41)	290 (15.25)	436 (22.92)	553 (29.09)	0.000	
		Diabetes	483 (6.35)	77 (4.05)	112 (5.89)	146 (7.68)	148 (7.79)	0.000	
		Dyslipidaemia	733 (9.64)	77 (4.05)	151 (7.94)	246 (12.93)	259 (13.62)	0.000	
	Menopausal status							0.000	
		No	1720 (52.89)	446 (66.67)	446 (59.79)	401 (49.26)	427 (41.74)		
		Yes	1532 (47.11)	223 (33.33)	300 (40.21)	413 (50.74)	596 (58.26)		
	Survey year							0.000	
		2008	1460 (19.19)	455 (23.92)	378 (19.87)	363 (19.09)	264 (13.89)		
		2009	2894 (38.04)	778 (40.90)	777 (40.85)	725 (38.12)	614 (32.30)		
		2010	2295 (30.17)	420 (22.08)	503 (26.45)	599 (31.49)	773 (40.66)		
		2011	958 (12.59)	249 (13.09)	244 (12.83)	215 (11.30)	250 (13.15)		
	MetS component								
		WC ≥ 85 cm	1902 (25.00)	75 (3.94)	248 (13.04)	574 (30.18)	1005 (52.87)	0.000	
		TG ≥ 150 mg/dL or drug treatment for dyslipidaemia	1565 (20.57)	202 (10.62)	342 (17.98)	488 (25.66)	533 (28.04)	0.000	
		HDL < 50 mg/dL	3936 (51.74)	794 (41.75)	929 (48.84)	1081 (56.83)	1132 (59.55)	0.000	
		BP ≥ 130/85 mmHg or drug treatment for elevated BP	1817 (23.89)	302 (15.88)	402 (21.14)	508 (26.71)	605 (31.83)	0.000	
		FPG ≥ 100 mg/dL or drug treatment for elevated FPG	1649 (21.68)	237 (12.46)	386 (20.29)	463 (24.34)	563 (29.62)	0.000	
	MetS (NCEP)		1635 (21.49)	143 (7.52)	291 (15.30)	494 (25.97)	707 (37.19)	0.000	
	MetS (IDF)		1156 (15.20)	48 (2.52)	145 (7.62)	356 (18.72)	607 (31.93)	0.000	
	HOMA‐IR		2.32 ± 0.02	1.90 ±0.03	2.17 ± 0.03	2.50 ± 0.05	2.75 ± 0.05	0.000	Q1 < Q2 < Q3 < Q4

Data are presented as means ± standard error for continuous variables and numbers (%) for categorical variables.

BP, blood pressure; FPG, fasting plasma glucose; HDL, high‐density lipoprotein; IPAQ, International Physical Activity Questionnaire; MetS, metabolic syndrome; NCEP, 2005 National Cholesterol Education Program Adult Treatment Panel III; IDF, 2006 International Diabetes Federation; TG, triglyceride; WC, waist circumference.

*P* value from linear regression analysis for continuous variables or *χ* ^2^ test for categorical variables, comparing differences among four groups.

Post‐hoc test in the linear regression analysis.

Categorical variable from the International Physical Activity Questionnaire research committee.

In men, the youngest age and highest proportion of low‐income participants were observed in Q1, and the highest proportion of highly educated participants was in Q4. In women, the youngest age and highest proportion of highly educated participants were in Q1, whereas income level did not significantly differ among the quartiles. In men, the daily total energy intake and water intake per body weight were the lowest in Q4, whereas the percentage of energy intake from protein was higher in Q4 compared with Q1. In women, the daily total energy intake and water intake per body weight were the lowest in Q4, and the energy intake from fat was lower in Q4 compared with Q1.

### 3.2 Correlations of the fat‐to‐muscle ratio with sex, age, and nutrient intake

**Figure 1:**
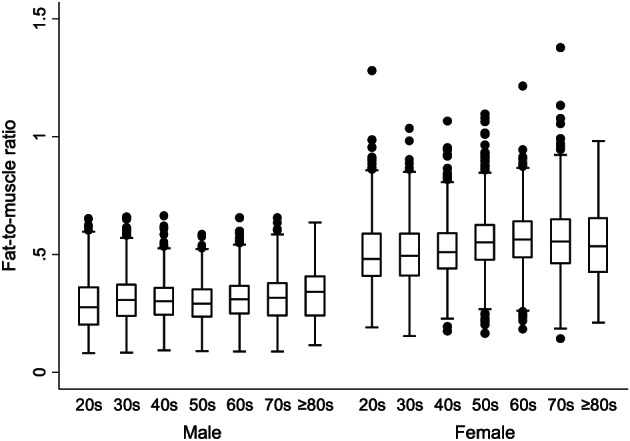
Fat‐to‐muscle ratio according to sex and age.

The FMR significantly differed between men and women at all ages (0.30 ± 0.002 vs. 0.53 ± 0.003, respectively, *P* < 0.001; *Figure* *1*). The highest mean FMR was observed in women in their 60s, whereas the FMR increased with age in men. In women, the daily total energy intake and the percentage of energy intake from carbohydrate decreased, whereas the percentage of energy intake from protein and fat increased, as FMR increased, after adjusting for potential confounding variables. On the other hand, in men, the daily total energy intake decreased, and the percentage of energy intake from fat increased, as FMR increased, after adjusting for potential confounding variables (*Table* 2).

**Table 2:** Beta coefficients for nutritional intake according to fat‐to‐muscle ratio quartile

Nutritional intake	Sex	Fat‐to‐muscle ratio quartile	Crude	Age adjusted	Multivariable^a^
Total energy, kcal/day					
	Male (*n* = 5425)				
		Q1 (*n* = 1357)	Reference	Reference	Reference
		Q2 (*n* = 1356)	−41.45 ± 41.40	−13.88 ± 41.06	−31.13 ± 40.84
		Q3 (*n* = 1356)	−56.43 ± 38.87	−24.04 ± 38.34	−48.13 ± 37.90
		Q4 (*n* = 1356)	−170.38 ± 42.43	−147.67 ± 42.27	−184.20 ± 41.88
		*P* for trend^b^	0.000	0.001	0.000
	Female (*n* = 7607)				
		Q1 (*n* = 1902)	Reference	Reference	Reference
		Q2 (*n* = 1902)	−39.69 ± 26.75	−23.38 ± 26.71	−31.78 ± 26.07
		Q3 (*n* = 1902)	−78.85 ± 26.93	−49.10 ± 26.98	−58.15 ± 26.69
		Q4 (*n* = 1901)	−125.72 ± 25.90	−91.05 ± 25.78	−108.67 ± 25.40
		*P* for trend^b^	0.000	0.000	0.000
Carbohydrate, % of energy					
	Male (*n* = 5425)				
		Q1 (*n* = 1357)	Reference	Reference	Reference
		Q2 (*n* = 1356)	0.78 ± 0.60	−0.05 ± 0.57	0.07 ± 0.57
		Q3 (*n* = 1356)	−0.75 ± 0.68	−1.72 ± 0.63	−1.37 ± 0.62
		Q4 (*n* = 1356)	−0.04 ± 0.65	−0.73 ± 0.62	−0.63 ± 0.60
		*P* for trend^b^	0.429	0.051	0.081
	Female (*n* = 7607)				
		Q1 (*n* = 1902)	Reference	Reference	Reference
		Q2 (*n* = 1902)	0.69 ± 0.48	−0.46 ± 0.45	−0.46 ± 0.43
		Q3 (*n* = 1902)	1.23 ± 0.48	−0.85 ± 0.44	−0.94 ± 0.44
		Q4 (*n* = 1901)	0.78 ± 0.49	−1.65 ± 0.44	−1.67 ± 0.42
		*P* for trend^b^	0.056	0.000	0.000
Protein, % of energy					
	Male (*n* = 5425)				
		Q1 (*n* = 1357)	Reference	Reference	Reference
		Q2 (*n* = 1356)	0.12 ± 0.18	0.22 ± 0.18	0.09 ± 0.18
		Q3 (*n* = 1356)	0.33 ± 0.20	0.45 ± 0.20	0.31 ± 0.19
		Q4 (*n* = 1356)	0.38 ± 0.19	0.46 ± 0.19	0.27 ± 0.20
		*P* for trend^b^	0.028	0.008	0.098
	Female (*n* = 7607)				
		Q1 (*n* = 1902)	Reference	Reference	Reference
		Q2 (*n* = 1902)	0.11 ± 0.16	0.26 ± 0.16	0.22 ± 0.16
		Q3 (*n* = 1902)	0.12 ± 0.15	0.39 ± 0.15	0.40 ± 0.15
		Q4 (*n* = 1901)	0.17 ± 0.15	0.50 ± 0.16	0.53 ± 0.15
		*P* for trend^b^	0.270	0.001	0.000
Fat, % of energy					
	Male (*n* = 5425)				
		Q1 (*n* = 1357)	Reference	Reference	Reference
		Q2 (*n* = 1356)	−0.45 ± 0.38	0.25 ± 0.35	−0.03 ± 0.34
		Q3 (*n* = 1356)	0.59 ± 0.39	1.42 ± 0.35	1.12 ± 0.36
		Q4 (*n* = 1356)	0.25 ± 0.40	0.83 ± 0.36	0.36 ± 0.36
		*P* for trend^b^	0.154	0.001	0.049
	Female (*n* = 7607)				
		Q1 (*n* = 1902)	Reference	Reference	Reference
		Q2 (*n* = 1902)	−0.64 ± 0.36	0.22 ± 0.34	0.22 ± 0.32
		Q3 (*n* = 1902)	−1.53 ± 0.34	0.03 ± 0.30	0.07 ± 0.30
		Q4 (*n* = 1901)	−1.06 ± 0.39	0.77 ± 0.36	0.82 ± 0.35
		*P* for trend^b^	0.001	0.059	0.035

Data are presented as beta coefficients ± standard error.

a Multivariable model adjusted for age, smoking, alcohol consumption, physical activity, education, income, and survey year.

b Test for linear trend across fat‐to‐muscle ratio quartiles.

### 3.3 Correlations of the fat‐to‐muscle ratio with metabolic syndrome and insulin resistance

**Figure 2:**
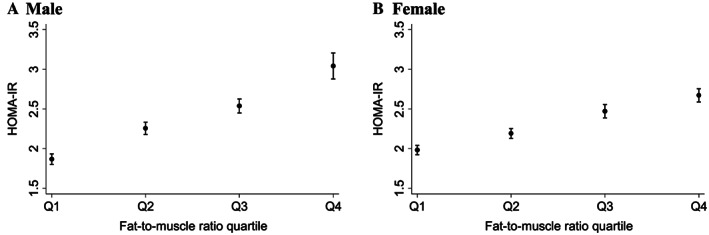
Least square mean (marginal mean) of the HOMA‐IR according to the fat‐to‐muscle ratio. Multivariable regression models were adjusted for age, energy intake, water intake per body weight, smoking, alcohol consumption, physical activity, education, income, and survey year. HOMA‐IR, homeostasis model assessment for insulin resistance. (A) Male; (B) Female.

There were significant differences in IR status according to FMR quartile (*Figure* *2*), in that the HOMA‐IR gradually increased as FMR increased, even after adjusting for potential confounding variables. The prevalence of MetS also gradually increased as FMR increased after adjusting for potential confounding variables (*P* for trend < 0.001; *Table* 3). The multivariable‐adjusted ORs for MetS (according to the IDF criteria) in FMR Q2, Q3, and Q4 compared with Q1 were 6.43 [95% confidence interval (CI): 3.98–10.40], 14.00 (95% CI: 8.69–22.57), and 36.26 (95% CI: 22.81–57.63), respectively, for men and 3.05 (95% CI: 2.02–4.62), 8.32 (95% CI: 5.72–12.12), and 17.63 (95% CI: 11.94–26.04), respectively, for women.

**Table 3:** Odds ratios for the risk of metabolic syndrome according to fat‐to‐muscle ratio quartile

MetS criteria	Sex	Quartile of fat‐to‐muscle ratio	Crude	Age adjusted	Multivariable^a^
NCEP					
	Male (*n* = 5425)				
		Q1 (*n* = 1357)	Reference	Reference	Reference
		Q2 (*n* = 1356)	3.79 (2.83 to 5.07)	3.62 (2.70 to 4.84)	3.77 (2.81 to 5.05)
		Q3 (*n* = 1356)	5.67 (4.30 to 7.49)	5.43 (4.12 to 7.15)	5.68 (4.29 to 7.52)
		Q4 (*n* = 1356)	9.55 (7.30 to 12.51)	9.82 (7.49 to 12.88)	11.01 (8.33 to 14.54)
		*P* for trend^b^	0.000	0.000	0.000
	Female (*n* = 7607)				
		Q1 (*n* = 1902)	Reference	Reference	Reference
		Q2 (*n* = 1902)	2.35 (1.79 to 3.09)	2.15 (1.63 to 2.84)	2.15 (1.62 to 2.86)
		Q3 (*n* = 1902)	4.85 (3.70 to 6.34)	4.03 (3.08 to 5.27)	3.96 (3.02 to 5.20)
		Q4 (*n* = 1901)	8.31 (6.39 to 10.81)	6.96 (5.32 to 9.11)	6.73 (5.13 to 8.82)
		*P* for trend^b^	0.000	0.000	0.000
IDF					
	Male (*n* = 5425)				
		Q1 (*n* = 1357)	Reference	Reference	Reference
		Q2 (*n* = 1356)	6.60 (4.10 to 10.63)	6.23 (3.87 to 10.03)	6.43 (3.98 to 10.40)
		Q3 (*n* = 1356)	14.21 (8.88 to 22.76)	13.42 (8.41 to 21.42)	14.00 (8.69 to 22.57)
		Q4 (*n* = 1356)	31.18 (19.83 to 49.04)	31.09 (19.81 to 48.80)	36.26 (22.81 to 57.63)
		*P* for trend^b^	0.000	0.000	0.000
	Female (*n* = 7607)				
		Q1 (*n* = 1902)	Reference	Reference	Reference
		Q2 (*n* = 1902)	3.43 (2.25 to 5.21)	3.09 (2.04 to 4.68)	3.05 (2.02 to 4.62)
		Q3 (*n* = 1902)	10.26 (6.99 to 15.07)	8.49 (5.85 to 12.32)	8.32 (5.72 to 12.12)
		Q4 (*n* = 1901)	21.93 (14.78 to 32.55)	18.31 (12.42 to 26.99)	17.63 (11.94 to 26.04)
		*P* for trend^b^	0.000	0.000	0.000

Data are presented as odds ratios (95% confidence interval).

IDF, 2006 International Diabetes Federation; NCEP, 2005 National Cholesterol Education Program Adult Treatment Panel III.

a Multivariable: adjusted for age, energy intake, water intake per body weight, smoking, alcohol consumption, physical activity, education, income, and survey year.

b Test for linear trend across fat‐to‐muscle ratio quartiles.

### 3.4 Cut‐off fat‐to‐muscle ratio for detecting metabolic syndrome

**Figure 3:**
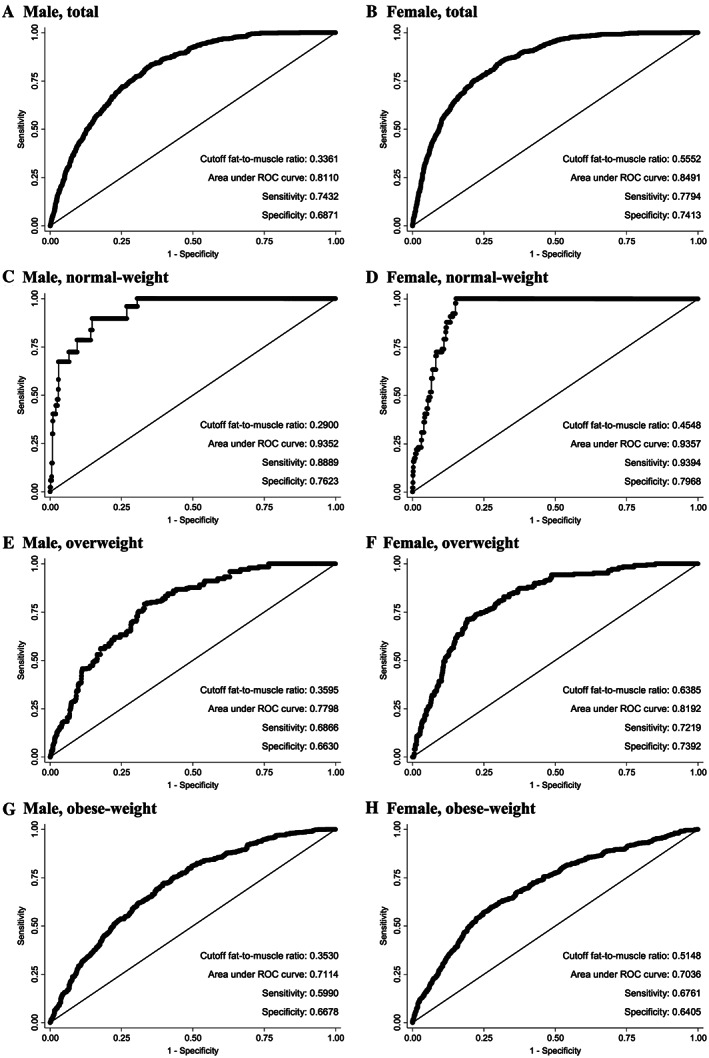
Cut‐off fat‐to‐muscle ratios for detecting metabolic syndrome ROC curves of the fat‐to‐muscle ratio for detecting metabolic syndrome (according to the IDF criteria) on the basis of multivariable logistic regression analysis. Multivariable regression models were adjusted for age, energy intake, water intake per body weight, smoking, alcohol consumption, physical activity, education, income, and survey year. IDF, 2006 International Diabetes Federation; ROC, receiver operating characteristic. (A) Male, total; (B) Female, total; (C) Male, normal weight; (D) Female, normal weight; (E) Male, overweight; (F) Female, overweight; (G) Male, obese weight; (H) Female, obese weight.

*Figure* *3* shows the ROC curves, cut‐off levels, and area under the ROC curve (AUC) values according to obesity status. Regardless of BMI, the cut‐off level was higher in women than in men (0.555 vs. 0.336 kg/kg, respectively). The sensitivity, specificity, and likelihood ratio positive were higher in women than in men (0.7794, 0.7413, 3.0126 vs. 0.7432, 0.6871, 2.3754, respectively), while the likelihood ratio negative was lower in women than in men (0.2976 vs. 0.3738). In normal‐weight participants, the sensitivity, specificity, and likelihood ratio positive were higher in women than in men (0.9394, 0.7968, 4.6223 vs. 0.8889, 0.7623, 3.7397, respectively), while the likelihood ratio negative was lower in women than in men (0.0761 vs. 0.1458). In overweight participants, the sensitivity, specificity, and likelihood ratio positive were higher in women than in men (0.7219, 0.7392, 2.7679 vs. 0.6866, 0.6630, 2.0375, respectively), while the likelihood ratio negative was lower in women than in men (0.3762 vs. 0.4727). In obese participants, the sensitivity, specificity, and likelihood ratio positive were higher in women than in men (0.6761, 0.6405, 1.8808 vs. 0.5990, 0.6678, 1.8031, respectively), while the likelihood ratio negative was lower in women than in men (0.5057 vs. 0.6005). The *C*‐statistic of FMR for MetS was the highest in normal‐weight participants (AUC: 0.9357 in women and 0.9352 in men), followed by overweight (AUC: 0.8192 in women and 0.7798 in men) and obese participants (AUC: 0.7036 in women and 0.7114 in men). The negative predictive value was the highest in normal‐weight participants (0.9992 in women and 0.9986 in men), while the positive predictive value was the highest in obese participants (0.5994 in women and 0.5428 in men).

Based on the derived cut‐off point, a high FMR was associated with poor outcomes for all cardiometabolic risk markers (*P* < 0.001; *Table* 4). All multivariable‐adjusted ORs of the cardiometabolic risk markers according to FMR were significant (*Table* 5). The multivariable‐adjusted ORs for MetS, WC, HOMA‐IR ≥ 2, and HOMA‐IR ≥ 3 were 5.35 (95% CI: 4.39–6.52), 7.67 (95% CI: 6.33–9.30), 3.00 (95% CI: 2.53–3.56), and 3.25 (95% CI: 2.70–3.92), respectively, in men and 5.59 (95% CI: 4.66–6.72), 7.48 (95% CI: 6.35–8.82), 2.32 (95% CI: 2.05–2.63), and 2.55 (95% CI: 2.17–3.00), respectively, in women.

**Table 4:** Sex‐specific thresholds of the fat‐to‐muscle ratio for detecting a high risk of metabolic syndrome according to the International Diabetes Federation criteria using anthropometric, blood pressure, and metabolic parameters

Characteristics		Male (*n* = 5425)			Female (*n* = 7607)		
		FMR < 0.3361079	FMR ≥ 0.3361079	*P* value^a^	FMR < 0.5552342	FMR ≥ 0.5552342	*P* value^a^
		(*n* = 3542)	(*n* = 1883)		(*n* = 4540)	(*n* = 3067)	
Anthropometric parameters							
Body weight, kg		66.92 ± 0.20	76.20 ± 0.37	0.000	54.19 ± 0.12	62.65 ± 0.25	0.000
Body mass index, kg/m^2^		22.97 ± 0.06	26.14 ± 0.11	0.000	21.65 ± 0.05	25.57 ± 0.09	0.000
Waist circumference, cm		80.82 ± 0.19	90.20 ± 0.29	0.000	73.63 ± 0.18	83.79 ± 0.27	0.000
Waist‐to‐height ratio		0.47 ± 0.001	0.53 ± 0.002	0.000	0.47 ± 0.001	0.54 ± 0.002	0.000
Fat mass, kg		12.69 ± 0.08	21.13 ± 0.14	0.000	16.60 ± 0.06	23.82 ± 0.11	0.000
Body fat, %		18.92 ± 0.09	27.83 ± 0.10	0.000	29.40 ± 0.08	38.15 ± 0.09	0.000
Fat mass index		0.44 ± 0.003	0.72 ± 0.004	0.000	0.64 ± 0.002	0.97 ± 0.005	0.000
FMR		0.25 ± 0.001	0.41 ± 0.002	0.000	0.44 ± 0.001	0.66 ± 0.003	0.000
Obesity status				0.000			0.000
	Underweight	179 (5.05)	1 (0.05)		394 (8.68)	14 (0.46)	
	Normal	1634 (46.13)	273 (14.50)		2726 (60.04)	648 (21.13)	
	Overweight	954 (26.93)	462 (24.54)		903 (19.89)	769 (25.07)	
	Obesity	775 (21.88)	1147 (60.91)		517 (11.39)	1636 (53.34)	
Blood pressure							
Systolic blood pressure		116.97 ± 0.34	120.13 ± 0.43	0.000	110.64 ± 0.34	117.07 ± 0.45	0.000
Diastolic blood pressure		76.64 ± 0.25	79.02 ± 0.34	0.000	71.23 ± 0.21	74.55 ± 0.27	0.000
Metabolic parameters							
Total cholesterol, mg/dL		183.38 ± 0.71	193.08 ± 1.09	0.000	180.34 ± 0.63	194.10 ± 0.77	0.000
TG, mg/dL		140.22 ± 2.52	180.80 ± 4.12	0.000	97.77 ± 1.24	123.05 ± 1.60	0.000
HDL, mg/dL		47.64 ± 0.25	43.32 ± 0.27	0.000	52.68 ± 0.22	49.12 ± 0.24	0.000
FPG, mg/dL		96.86 ± 0.46	100.69 ± 0.68	0.000	92.67 ± 0.33	96.74 ± 0.45	0.000
HOMA‐IR		2.13 ± 0.03	2.97 ± 0.07	0.000	2.10 ± 0.02	2.67 ± 0.04	0.000
MetS (IDF)		317 (8.95)	598 (31.76)	0.000	302 (6.65)	854 (27.84)	0.000

Data are presented as means ± standard error for continuous variables and numbers (%) for categorical variables.

FMR, fat‐to‐muscle ratio; FPG, fasting plasma glucose; HDL, high‐density lipoprotein; HOMA‐IR, homeostasis model assessment of insulin resistance; IDF, 2006 International Diabetes Federation; MetS, metabolic syndrome; TG, triglyceride.

a *P* value from linear regression analysis for continuous variables or *χ* ^2^ test for categorical variables, comparing differences between two groups.

**Table 5:** Odds ratios for each outcome variable according to the fat‐to‐mass ratio (by cut‐off level)

Sex	Outcome variables (dependent variable)	Crude	Age adjusted	Multivariable^a^
Male (*n* = 5425)				
	MetS (IDF)	5.00 (4.14 to 6.03)	5.05 (4.19 to 6.09)	5.35 (4.39 to 6.52)
	WC ≥ 90 cm	6.98 (5.86 to 8.31)	7.10 (5.97 to 8.44)	7.67 (6.33 to 9.30)
	Total cholesterol ≥ 200 mg/dL	1.57 (1.36 to 1.82)	1.56 (1.34 to 1.80)	1.52 (1.30 to 1.77)
	TG ≥ 150 mg/dL or drug treatment for dyslipidaemia	2.30 (2.01 to 2.63)	2.29 (1.99 to 2.62)	2.34 (2.02 to 2.70)
	HDL < 40 mg/dL	1.89 (1.65 to 2.16)	1.87 (1.63 to 2.15)	1.86 (1.62 to 2.14)
	BP ≥ 130/85 mmHg or drug treatment for elevated BP	1.61 (1.42 to 1.84)	1.60 (1.39 to 1.84)	1.63 (1.41 to 1.89)
	FPG ≥ 100 mg/dL or drug treatment for elevated FPG	1.59 (1.38 to 1.84)	1.59 (1.38 to 1.84)	1.66 (1.42 to 1.94)
	HOMA‐IR ≥ 2	3.27 (2.77 to 3.86)	3.27 (2.77 to 3.86)	3.00 (2.53 to 3.56)
	HOMA‐IR ≥ 3	3.56 (2.97 to 4.27)	3.54 (2.96 to 4.24)	3.25 (2.70 to 3.92)
Female (*n* = 7607)				
	MetS (IDF)	6.34 (5.31 to 7.56)	5.72 (4.79 to 6.83)	5.59 (4.66 to 6.72)
	WC ≥ 85 cm	7.61 (6.49 to 8.93)	7.23 (6.16 to 8.47)	7.48 (6.35 to 8.82)
	Total cholesterol ≥ 200 mg/dL	2.02 (1.79 to 2.26)	1.75 (1.55 to 1.99)	1.74 (1.54 to 1.97)
	TG ≥ 150 mg/dL or drug treatment for dyslipidaemia	2.18 (1.90 to 2.49)	1.85 (1.61 to 2.13)	1.82 (1.58 to 2.10)
	HDL < 50 mg/dL	1.72 (1.54 to 1.92)	1.56 (1.39 to 1.75)	1.52 (1.36 to 1.71)
	BP ≥ 130/85 mmHg or drug treatment for elevated BP	1.97 (1.71 to 2.27)	1.55 (1.32 to 1.82)	1.51 (1.29 to 1.78)
	FPG ≥ 100 mg/dL or drug treatment for elevated FPG	1.89 (1.64 to 2.17)	1.56 (1.35 to 1.80)	1.56 (1.35 to 1.79)
	HOMA‐IR ≥ 2	2.61 (2.32 to 2.95)	2.52 (2.22 to 2.85)	2.32 (2.05 to 2.63)
	HOMA‐IR ≥ 3	3.00 (2.57 to 3.51)	2.76 (2.36 to 3.24)	2.55 (2.17 to 3.00)

Data are presented as odds ratios (95% confidence interval).

BP, blood pressure; FPG, fasting plasma glucose; HDL, high‐density lipoprotein; HOMA‐IR, homeostasis model assessment of insulin resistance; IDF, 2006 International Diabetes Federation; MetS, metabolic syndrome; TG, triglyceride; WC, waist circumference.

a Multivariable model adjusted for age, energy intake, water intake per body weight, smoking, alcohol consumption, physical activity, education, income, and survey year.

## 4 Discussion

The present analysis of nationally representative survey data from the KNHANES revealed that high FMR was significantly associated with the prevalence of MetS as well as the components of MetS and IR. In addition, the negative predictive value was particularly high in normal‐weight participants, while the positive predictive value was particularly high in obese participants. This is the first large cross‐sectional study to investigate the associations of FMR (assessed by DXA) with MetS and IR and to determine sex‐specific optimal cut‐off values of FMR for predicting MetS and IR in a Korean population.

Although a high BMI has been consistently associated with cardiometabolic risks and is often used as a simple indicator of obesity in large populations, its limitations as an index of obesity are well established. For example, Asian populations have lower BMI values, but a higher risk of IR as well as a higher visceral fat level or fat percentage for a given BMI, than have Caucasians.^10^, ^11^, ^40^ Many studies have investigated the associations of skeletal muscle mass and FM or %BF with MetS in Asian populations.^21^, ^24^, ^25^, ^41^, ^42^ Muscle mass and strength are protective factors against cardiometabolic risk,^24^, ^25^, ^26^, ^43^ whereas high FM index, %BF, and visceral obesity are each positively associated with MetS and IR.^27^, ^44^, ^45^ In addition, a decrease in muscle mass can reduce the basal metabolic rate and PA, which can subsequently lead to an increase in FM. Although BMI is positively associated with FM and skeletal muscle mass, an increase in FM may occur simultaneously with loss of skeletal muscle mass in the absence of weight gain.^24^ The double burden of excess FM and low muscle mass can lead to higher risks of MetS and IR.^42^

According to the metabolic load‐capacity model, FMR may be a potential indicator of the combined effects of FM and skeletal muscle mass.^29^ Because FMR may be a more appropriate index for assessing cardiometabolic risk than are indices of each individual component, several studies have examined the clinical utility of the FMR.^28^, ^30^, ^31^, ^32^, ^33^, ^46^, ^47^, ^48^ Recently, the FMR has been used as a novel indicator of MetS.^28^, ^46^ Those studies measured body composition using BIA in a Colombian cohort of 1416 young subjects and determined the optimal cut‐off FMR for detecting MetS in a young population. Similarly, Xu *et al*.^46^ showed that FMR measured by BIA had a high predictive power for MetS in a Chinese population. In the present study, body composition was measured by DXA, which is considered a reference method; and the optimal cut‐off FMR values for the detection of MetS on the basis of the IDF criteria were 0.336 kg/kg in men and 0.555 kg/kg in women. Variations among these studies may be the result of racial and ethnic differences and/or the methods used to assess body composition, because BIA tends to underestimate FM but overestimate muscle mass. In a population of 6256 Korean subjects, Park *et al*.^48^ found that the muscle‐to‐fat ratio is a useful indicator for the management and early prevention of MetS. In the present study, the dietary information, alcohol and smoking habits, socio‐economic status, and co‐morbidities of 13 032 participants were assessed to investigate the associations of FMR with MetS and IR, and the results supported the hypothesis that FMR is a useful indicator for screening MetS and IR.

The present study also found that the highest average FMR in women occurred when they are in their 60s, whereas the FMR increased with age in men. Aging is associated with detrimental changes in body composition, and changes in fat distribution and body composition are accelerated by the menopausal transition in women. Furthermore, age‐related increases in FM may simultaneously occur with the loss of skeletal muscle mass in the absence of weight gain, and these changes can result in an increased FMR with aging. The present findings confirmed that a high FMR was associated with MetS and IR and showed that energy intake was associated with the FMR. In men, the daily total energy intake was the lowest in FMR Q4, but the percentage of energy intake from protein was higher in Q4 compared with Q1. In women, the daily total energy intake was the lowest in FMR Q4. Because skeletal muscle mass is responsible for an important portion of total energy expenditure, sufficient calories and nutrients might contribute to the preservation of skeletal muscle mass. For example, there is a positive association between daily energy intake and skeletal muscle mass,^49^, ^50^ and dietary intake is related to MetS in that the Western diet is associated with the development of MetS. Although traditional Korean meals contain high proportions of vegetables and carbohydrates with low proportions of fat, previous findings of the association between diet and MetS in this population have been inconclusive.^51^, ^52^, ^53^, ^54^ Similar to the present results, Woo *et al*.^53^ found a positive association between meat consumption and MetS but only in men; the sex difference may be related to the relatively low red meat consumption in women compared with men. Likewise, Kim *et al*.^54^ suggested that the relatively low consumption of red meat by Asians compared with Caucasians can explain these inconsistent results. However, after potential confounding variables were adjusted for, the daily total energy intake and the percentage of energy intake from carbohydrate decreased, whereas the percentage of energy intake from protein and fat increased, as FMR increased, in women. In men, the daily total energy intake decreased, and the percentage of energy intake from fat increased, as FMR increased, after adjusting for potential confounding variables. A recent study in mice reported that only increased dietary fat intake, but not protein or carbohydrate intake, causes adiposity.^55^ A recent randomized controlled trial found that changes in the percentage of energy intake from fat correlated positively with changes in FM, even after adjustment for changes in BMI and energy intake.^56^ Moreover, dietary fat restriction results in greater loss of body fat than does carbohydrate restriction in people with obesity.^57^ Therefore, in this study, it is expected that fat intake would have played a major role in the increase in FMR, even considering the influence of other factors.

In the present study, the proportion of participants with a low PA and the prevalence of hypertension, diabetes, and dyslipidaemia increased according to the FMR quartile. Although PA did not differ among the four FMR quartiles in younger women (<50 years of age), the proportion with low PA increased with the FMR quartile in women ≥ 50 years of age. The close relationships between high PA level and high lean mass and low %BF are well documented.^58^

The present study had several limitations that should be considered. The analyses were conducted using data from the KNHANES questionnaires, but the primary study variables were measured directly in all participants. Furthermore, this cross‐sectional study had a limited ability to demonstrate causal relationships between MetS and IR and other variables. However, the present results can be generalized to all Koreans owing to the large population sample size, the high response rate (~80%), and the use of proportional systematic sampling with multistage stratification based on geographical area, sex, and age group. This study also has strengths: the use of a large‐scale nationally representative dataset and the measurement of body composition using DXA, which is the gold standard, rather than BIA. In addition, the present analyses were adjusted for dietary information, lifestyle factors, socio‐economic status, and co‐morbidities. Finally, the present study was also able to determine differences in the predictive nature of the FMR according to BMI.

## 5 Conclusions

In conclusion, a high FMR was significantly associated with the prevalence of MetS and IR. In addition, the negative predictive value was particularly high in normal‐weight participants, while the positive predictive value was particularly high in obese participants. Therefore, FMR can be used as a novel indicator for detecting the absence or presence of MetS, particularly in metabolically healthy normal‐weight individuals and metabolically obese obese‐weight individuals. This is the first large cross‐sectional study to investigate the associations of FMR, as assessed by DXA, with MetS and IR and to determine sex‐specific optimal cut‐off values of the FMR for predicting MetS and IR in the Korean population.

## Author Contributions

Y.‐G. S. and Y. R. S. wrote the manuscript; Y.‐G. S. analysed the data; Y.‐G. S., H. J. S., and Y. R. S. interpreted the results; H. J. S. and Y. R. S. participated in the design of the study. All authors reviewed the manuscript, contributed to the discussion, and read and approved the final manuscript.

## Conflict of Interest

The authors declare no conflict of interest.
